# Instability of the Northeast Greenland Ice Stream over the last 45,000 years

**DOI:** 10.1038/s41467-018-04312-7

**Published:** 2018-05-14

**Authors:** Nicolaj K. Larsen, Laura B. Levy, Anders E. Carlson, Christo Buizert, Jesper Olsen, Astrid Strunk, Anders A. Bjørk, Daniel S. Skov

**Affiliations:** 1 Department of Geoscience, Aarhus University, Aarhus, 8000 Denmark; 2 Centre for GeoGenetics, Natural History Museum, University of Copenhagen, Copenhagen, 1350 Denmark; 3 Department of Geology, Humboldt State University, Arcata, 95521 CA USA; 4 College of Earth, Ocean, and Atmospheric Sciences, Oregon State University, Corvallis, 97331 OR USA; 5 Department of Physics and Astronomy, Aarhus University, Aarhus, 8000 Denmark; 6 Department of Earth System Science, University of California, Irvine, 92697 CA USA

## Abstract

The sensitivity of the Northeast Greenland Ice Stream (NEGIS) to prolonged warm periods is largely unknown and geological records documenting such long-term changes are needed to place current observations in perspective. Here we use cosmogenic surface exposure and radiocarbon ages to determine the magnitude of NEGIS margin fluctuations over the last 45 kyr (thousand years). We find that the NEGIS experienced slow early Holocene ice-margin retreat of 30–40 m a^−1^, likely as a result of the buttressing effect of sea-ice or shelf-ice. The NEGIS was ~20–70 km behind its present ice-extent ~41–26 ka and ~7.8–1.2 ka; both periods of high orbital precession index and/or summer temperatures within the projected warming for the end of this century. We show that the NEGIS was smaller than present for approximately half of the last ~45 kyr and is susceptible to subtle changes in climate, which has implications for future stability of this ice stream.

## Abstract (web summary)

The outlet glaciers that comprise the Northeast Greenland Ice Stream (NEGIS) have experienced accelerated retreat in recent years, yet their longterm stability remains unclear. Here, via cosmogenic surface exposure and radiocarbon ages, the authors investigate the stability of the NEGIS for the past 45 kyr.

## Introduction

**Fig. 1:**
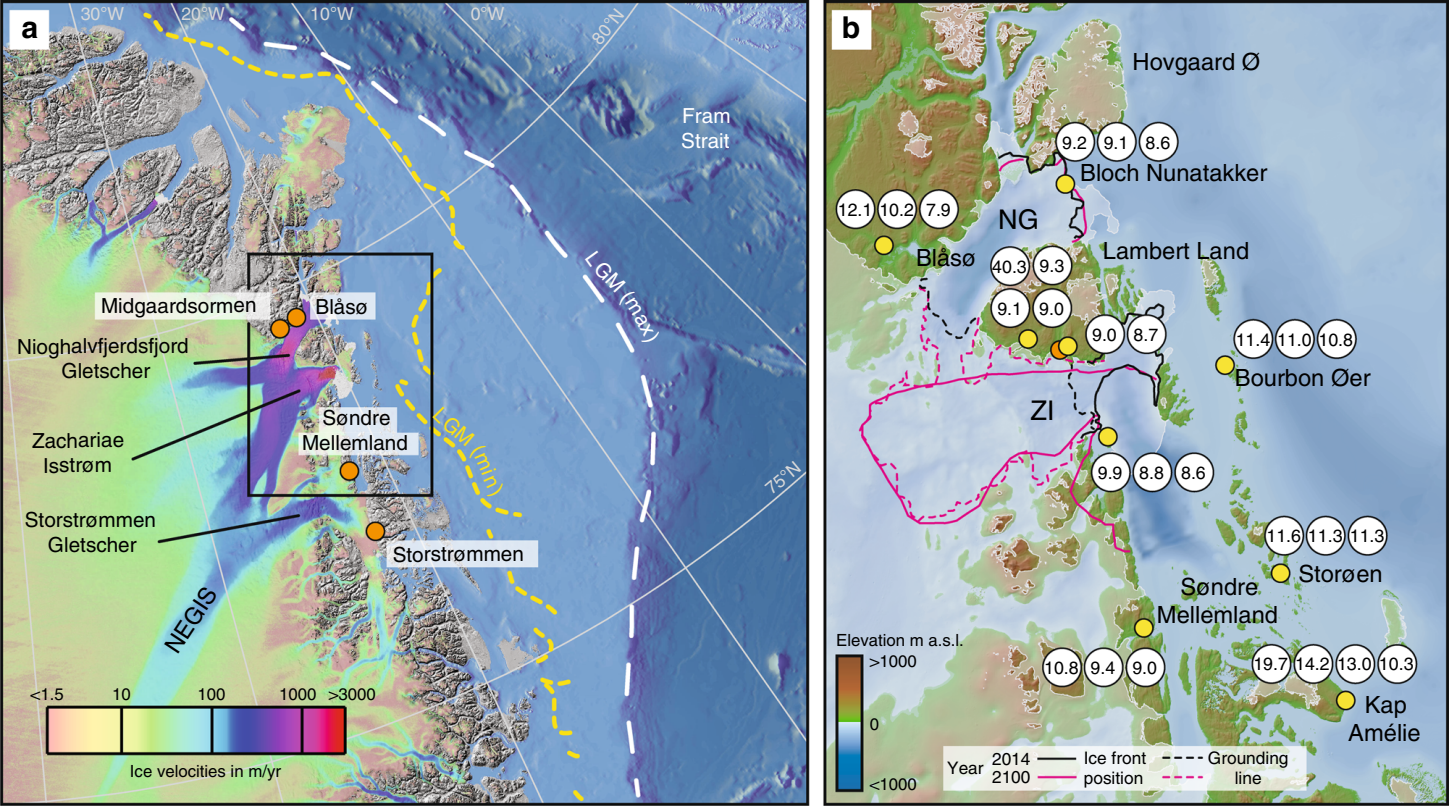
Field location of the Northeast Greenland Ice Stream. **a** Map with minimum and maximum LGM ice extent^8^, and velocity map of the inland ice^69^. Orange circles are sites with existing ^14^C dates reworked in Little Ice Age moraines or from raised marine deposits presently dammed by NEGIS^27,28^. **b** Inset map with bed topography, merged from existing topographic and bathymetric data with mass conservation (beneath grounded ice) and gravity inversion (beneath floating ice and open ocean)^70^. Outline of glacier margin is based on GIMP data^71^. The observed and modeled ice front and grounding line positions in 2014 C.E. and under maximum melting scenarios in 2100 C.E.^7^. New cosmogenic surface exposure ages (yellow circles) in ka (thousand years ago) from outer coast and proximal to the present ice margin in Northeast Greenland 78°N to 80°N. Red circles mark new ^14^C dates of reworked shell fragments from a moraine on Lambert Land

Greenland Ice Sheet (GrIS) mass loss has doubled since the beginning of the 20th century^1^. A prominent feature of the GrIS is the Northeast Greenland Ice Stream (NEGIS)^2,3^, which constitutes an ~600-km-long ice stream that drains ~12% of the interior GrIS via three fast-flowing marine-terminating outlet glaciers: Nioghalvfjerdsfjord Gletscher (NG), Zachariae Isstrøm (ZI), and Storstrømmen Gletscher (SG) (Fig. 1). From 2006 to 2012, the NG and ZI accelerated and retreated after more than a decade of stability^4^. ZI accelerated further in 2012 when its ice velocity tripled, losing its residual ice shelf^5^. Presently, ZI is rapidly retreating along a reverse-sloped marine-based bed, whereas NG is retreating slower along an upward-sloping bed^5^. In contrast, SG is at present relatively stable and in a phase of buildup^4^ following its 1978–1984 surge^6^. Modeling studies of NEGIS using different warming scenarios suggest that NG will not change significantly, whereas ZI will continue fast and unstoppable retreat 30 km upstream of its current position, contributing ~16.2 mm to global-mean sea-level rise by 2100 C.E.^7^. Then the ice margin will stabilize on a topographic ridge unless frontal summer melt rates exceed 6 m day^−1^, which would trigger further inland retreat; this is a forcing that is within the range of the possible Intergovernmental Panel on Climate Change (IPCC) scenarios^7^. To assess these modern observations and modeling scenarios, as well as the possibilities of future NEGIS collapse, a long-term perspective is urgently needed to understand the (in)stability of the NEGIS.

During the global Last Glacial Maximum (LGM; ~26–19 ka), the GrIS reached the continental shelf in Northeast Greenland, but it has been contentious whether it reached the shelf edge ~250–350 km from the present ice margin or remained on the inner shelf (Fig. 1a)^8^. High-resolution multibeam swath bathymetry and shallow seismic data from the shelf offshore of the NEGIS show a number of glacial landforms including mega-scale glacial lineations suggesting that the ice sheet margin extended to the shelf edge in Northeast Greenland^9–12^. The age of the landforms is, however, poorly constrained due to a lack of marine sediment cores from the area. It is assumed that they were formed during the LGM as they appear fresh and are not draped with significant sediment cover. Farther north at 81°N, the reduction in ice-rafted debris and lower sedimentation rates in marine sediment cores suggest that deglaciation from the shelf began ~20 ka^13^, whereas the shelf areas at Kejser Franz Josef Fjord (72°N) and Scoresby Sund (70°N) ~700–1000 km farther south experienced regional deglaciation ~17–19 ka^8,14–17^. Radiocarbon dates of postglacial marine shells from the coastline outside NEGIS indicate deglaciation around 9.7–9.1 ka; the later deglaciation of this area compared to adjacent areas to the north and south has been used to suggest more extensive glaciation reaching the shelf edge^18^. However, these ^14^C dates are only minimum-limiting ages, which are often significantly younger than ^10^Be ages of deglaciation in Greenland^19^. These limitations highlight the need for better age constraints to resolve the long-term ice-margin fluctuations of NEGIS.

Here we combine cosmogenic surface exposure ages (^10^Be) on glacial boulders with radiocarbon dates (^14^C) from reworked marine shells in moraines to reconstruct the last 45 kyr of NEGIS-margin fluctuations (see Methods). In addition, we investigate the climate forcings that may drive long-term ice-margin variability on these time scales. We find that the NEGIS experienced slow early Holocene ice-margin retreat of 30–40 m a^−1^ from the outer coast to the present ice margin, likely as a result of the buttressing effect of sea or shelf ice. We furthermore show that the NEGIS was smaller than present ~41–26 ka and ~7.8–1.2 ka, or approximately half of the last ~45 kyr as a result of air and ocean temperature forcings similar to or slightly higher than present.

## Results

### The timing of early Holocene ice retreat

Cosmogenic exposure dating is a widely used method to constrain the timing of deglaciation in Greenland^19–25^. In this study, we use twenty-eight ^10^Be boulder ages to constrain the NEGIS retreat from the coastline to the present-day ice margin (60–100 km) in Northeast Greenland (77.4–79.4°N) during the last deglaciation (Fig. 1a, b). By assuming that the deglaciation of the outer coast to present ice margin was largely synchronous within the study site, we calculated the mean and standard error of the mean for ^10^Be ages at the coast and present ice margin (Methods and Supplementary Table 1 and Table 2). At the three coastal sites on Bourbon Øer, Storøen, and Kap Amélie, ten samples were dated. After excluding one outlier, we calculated a mean ^10^Be age of 11.7 ± 0.4 (0.6) ka (uncertainty in parentheses includes the production rate uncertainty). Adjacent to the present ice margins at Blåsø, Lambert Land, ZI, Søndre Mellemland, and Bloch Nunatakker we dated eighteen samples. With one outlier excluded, we obtained a mean deglaciation age of 9.3 ± 0.2 (0.4) ka. Our ^10^Be ages demonstrate that the deglaciation of the outer coast to present ice margin, a distance of 60–100 km, was completed within ~2.4 kyr at an average retreat rate of 30–40 m a^−1^.

### The response of the NEGIS to a warmer climate

**Fig. 2:**
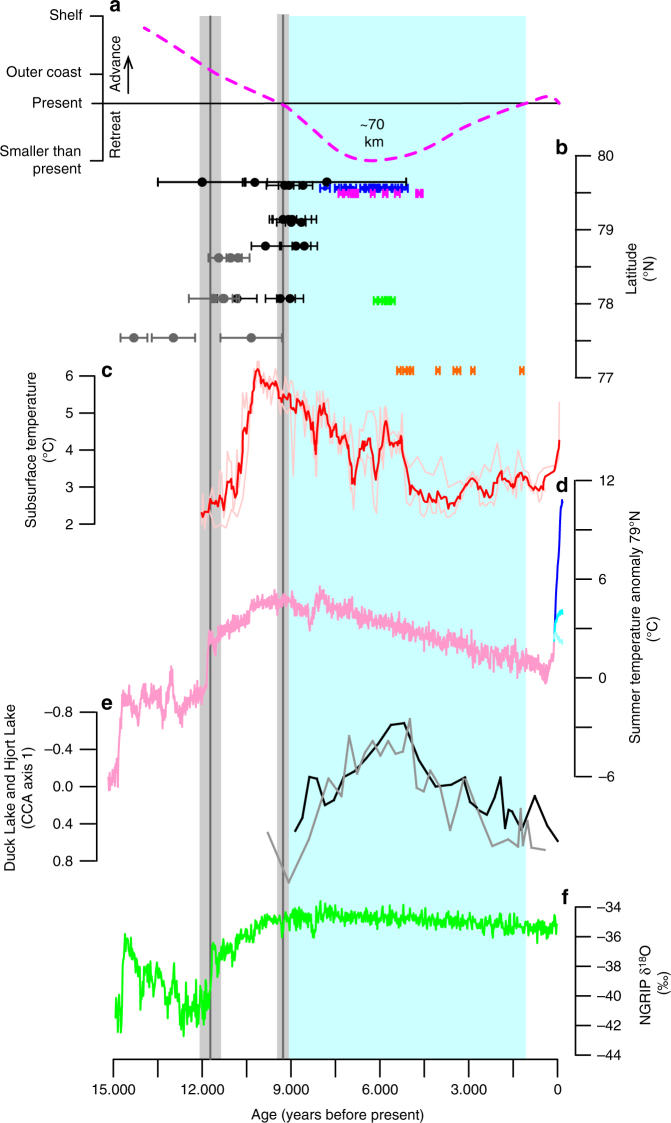
Ice fluctuations and climate variability the last 15 kyr. **a** Reconstruction of ice-margin fluctuations based on **b** ^10^Be ages with external uncertainties from outer coast (dark gray) and outside the Little Ice Age moraine (black), and ^14^C dates from raised marine deposits presently dammed by NEGIS up to 70 km upstream the present ice margin at NG; Blåsø (blue)^27^ and Midgaardsormen (pink)^27^, and ^14^C dates of reworked shells in Little Ice Age moraines at Søndre Mellemland (green)^27^, and at SG (orange)^28^. Vertical gray bars are the mean and standard error of the outer (older) and inner (younger) ^10^Be ages with the production rate uncertainty included. Vertical light blue bar is when the NEGIS is smaller than present. The early Holocene ice-retreat coincides with **c** peak subsurface temperatures based on planktic foraminiferal fauna assemblages (SST100)^32^ and **d** peak summer temperature (JJA) (pink) at 79°N with RCP 2.6 (light blue), 4.5 (blue), and 8.5 scenarios (dark blue)^31^. **e** The timing of maximum mid-Holocene ice retreat coincides with local chironomid-based temperature maxima at Duck (black) and Hjort (grey) lakes on Store Koldeway in Northeast Greenland^39^. **f** Ice core δ^18^O record from NGRIP^72^

**Fig. 3:**
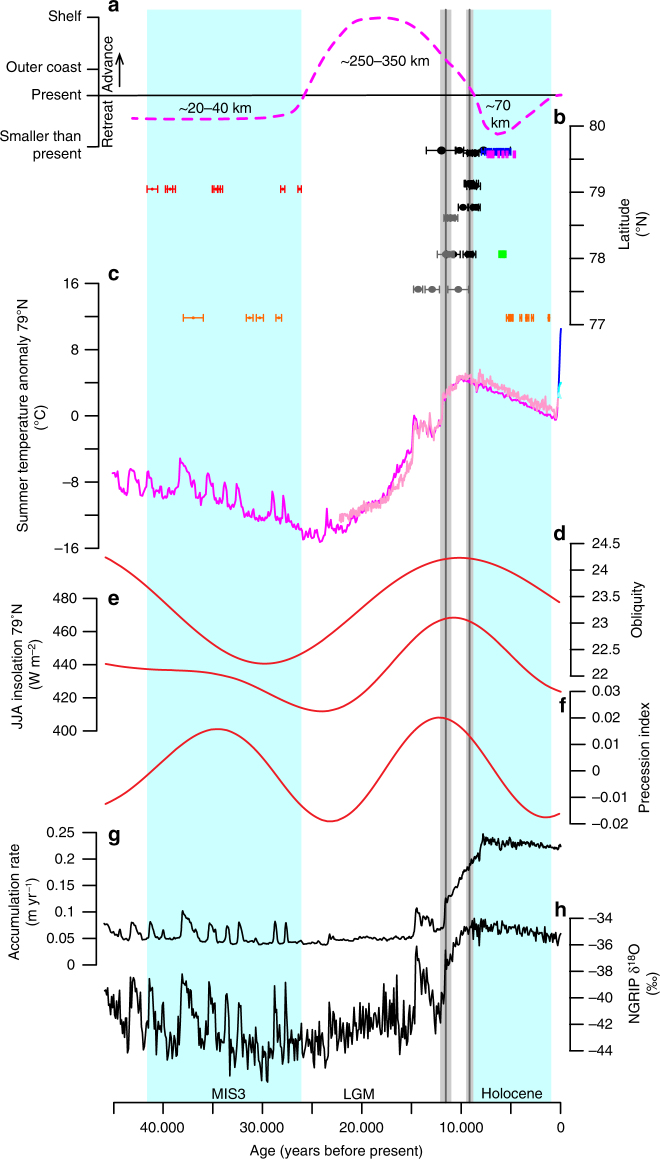
Ice and climate fluctuations of the Northeast Greenland Ice Stream for the last 45 kyr. **a** Reconstruction of ice-margin fluctuations based on **b** ^10^Be ages with external uncertainties from outer coast (dark gray) and outside the Little Ice Age moraine (black), and ^14^C dates from raised marine deposits presently dammed by NEGIS up to 70 km upstream the present ice margin at NG; Blåsø (blue)^27^ and Midgaardsormen (pink)^27^, and ^14^C dates of reworked shells in Little Ice Age moraines at Søndre Mellemland (green)^27^, and at SG (orange)^28^. Vertical gray bars are the mean and standard error of the outer (older) and inner (younger) ^10^Be ages with the production rate uncertainty included. Vertical light blue bars are when the NEGIS is smaller than present. **c** Reconstructed summer temperature (pink) with RCP 2.6 (light blue), 4.5 (blue), and 8.5 scenarios (dark blue) at 79°N^31^ and a summer temperatures reconstruction (magenta) based on a multiple-regression method (see Methods). **d**–**f** Orbital parameters and summer insolation at 79°N. **g** Accumulation rate at NEEM^36^. **h** Ice core δ^18^O record from NGRIP^72^

Radiocarbon dating of reworked marine material (shells or whale bones) in moraines is a method often used to infer times when the ice margins in Greenland were further inland than at present^26–29^. We have combined new and existing ^14^C dates from NEGIS’s three marine terminating outlet glaciers in the study area (Fig. 1a, b). Overall, there are two periods where the ^14^C dates show that the NEGIS was smaller than present during Marine Isotope Stage 3 (MIS 3) and the Holocene (Figs. 2 and 3). We present eight new ^14^C dates from Lambert Land adjacent to ZI ranging from 41.1 ± 0.5 to 26.3 ± 0.2 ka (Fig. 3b, Supplementary Table 3) that show that ZI retreated at least ~20 km inland from its 2014 position opening up for marine conditions farther inland. Four existing ^14^C dates from moraines adjacent to SG show that it was smaller than present at least 37.0 ± 1.0 to 28.4 ± 0.3 ka and open marine conditions extended at least ~40 km farther inland relative to present^28^. Based on the distribution of ^14^C ages from ZI and SG, it can be concluded that the NEGIS was continually retracted at least 20–40 km behind the present ice extent prior to ~41 ka and until after ~26 ka. Previously published radiocarbon dates of reworked shells and whale bones in Little Ice Age moraines and raised marine deposits likewise suggest that NEGIS was ~20–70 km farther inland than today ~7.8–1.2 ka, before it advanced to its Little Ice Age maximum in the 19th century^27,28^.

## Discussion

Our new ^10^Be ages demonstrate that deglaciation of the outer coast to the present ice margin occurred between 11.7 ± 0.6 and 9.3 ± 0.4 ka. This is ~2 kyr older than the oldest recorded ^14^C dates of ~9.7 ka from the outer coast^18^. The timing of early Holocene NEGIS retreat from the coast to its current extent generally coincides with both increased surface air and subsurface ocean temperatures (Fig. 2a–d). Summer surface air temperature reconstructed by merged ice-core data and climate models shows an abrupt rise in temperature at the Younger Dryas termination, coeval with the ^10^Be ages presented here for initial ice retreat from the coastline, followed by gradual warming to peak temperatures ~10–9 ka (Fig. 2d)^30,31^. In the Fram Strait, subsurface ocean warming is recorded slightly later ~10.8–10.1 ka, at a rate of ~0.5 °C per century and a peak Holocene temperature of ~6 °C at ~10.1 ka^32^ (Fig. 2c). We suggest that the combined effect of insolation-driven atmospheric/oceanic warming and abrupt atmospheric warming at the end of the Younger Dryas likely triggered NEGIS coastal to inland retreat.

The mean ^10^Be ages suggest that the deglaciation from the outer coast to the present ice margin occurred at a retreat rate of 30–40 m a^−1^. This estimate is slower than early Holocene retreat rates of ~100 m a^−1^ for Jakobshavn Isbræ in West Greenland^25^ and ~80 m a^−1^ for Helheim Glacier in Southeast Greenland^21^. It is striking that the marine-based parts of NEGIS only experienced moderate rates of ice retreat compared to other major GrIS outlet glaciers^21,25^. We attribute this to the buttressing effect of sea ice or shelf ice^33^, which was hinged on to islands off the coast in Northeast Greenland (Fig. 1) until ~9.5 ka^34,35^.

The ^14^C ages of reworked shells demonstrate that the NEGIS was retracted ~20–40 km during MIS 3 from ~41 to ~26 ka when ice-core data indicate that the mean-annual temperature was generally as cold as the LGM and accumulation rates (and therefore ice flow velocities) were 4–5 times slower than during the Holocene^36^ (Fig. 3g, h). However, as glaciers respond primarily to summer air temperatures^37^, we estimate local summer air temperatures at our site during MIS 3 using a multiple-regression method (see Methods). Approximately 41–26 ka estimated summer temperatures were ~6–8 °C warmer than the LGM primarily because of higher boreal summer insolation, but ~8–12 °C colder than the preindustrial period (Fig. 3c). The combination of relatively mild summers and low snow accumulation rates (Fig. 3c, g) seems to be a plausible explanation for the retracted NEGIS margin during MIS 3. The ^14^C dates from MIS 3 also provide a maximum age constraint for when the NEGIS began its advance toward its LGM position and show that the GrIS in this sector was larger than present after ~26 ka until 9.3 ± 0.4 ka (uncertainty including the production rate uncertainty), when the ^10^Be ages indicate that the areas in front of the present ice margin were again deglaciated. The timing of maximum LGM extent of NEGIS agrees with a Northeast Greenland marine record that places the ice margin on the continental shelf ~26–20 ka^13^.

Published ^14^C dates of marine shells and whale bones from the left-lateral margin of NG show that the floating ice margin was smaller than present extent and reached a minimum of at least 70 km behind its present extent ~7.8–4.6 ka^27^. At SG, reworked shells in Little Ice Age moraines suggest that it was smaller than present ~5.4–1.2 ka^28^. The timing of the retracted ice margin of the NEGIS outlet glaciers ~7.8–1.2 ka generally agrees with a smaller than present GrIS extent during the mid-Holocene thermal maximum seen in southern Greenland^38^. The timing of retracted NEGIS also coincides with local chironomid-based temperature maxima ~8–5 ka from Store Koldeway in Northeast Greenland^39^ and warm subsurface temperatures in Fram Strait^32^ (Fig. 2c, e). However, it is also concurrent with relatively high accumulation rates^36^, suggesting that the forcing of the mid-Holocene NEGIS retreat differs from that of MIS 3. Both periods of retracted NEGIS margins occurred during, or just after, periods of high orbital precession index (Fig. 3d–f), supporting the notion that precession forcing dominates the ice-sheet response on orbital time scales.

We compare our results with a recent state-of-the art modeling study, which suggests that the NG is difficult to destabilize when compared to the ZI and that bed topography plays a critical role in determining ice-margin responses to ocean warming^7^. The three-dimensional ice-sheet model is forced by constant surface mass balance and variable ocean forcing. It predicts that NG will likely keep its current configuration with the grounding line and the ice front close to its present location by 2100 C.E., even when the model is forced with high basal-melt rates and frontal-melt rates (Fig. 1b). Conversely, ZI is modeled to be more sensitive, with the simulations showing ~30 km ice-margin retreat before stabilization on a topographic ridge by 2100 C.E. unless frontal summer melt rates exceed 6 m day^−1^. This would trigger further inland retreat and the oceanic forcing is within the range of possible future scenarios^7^. The geologic data clearly demonstrate that both NG and ZI, as well as SG, retreated behind their present extents during the Holocene. These observations suggest that the model underestimates the sensitivity of NEGIS, and particularly the NG, to increased oceanic forcing. However even when the grounding line and ice velocity are stable, once the ice margin reaches a topographic ridge neither NG nor ZI reach a steady state and still lose mass over the entire duration of the simulation^7^. They could eventually retreat further inland on a longer time scale as demonstrated by the geological record.

In conclusion, we show that the NEGIS experienced major ice-margin fluctuations over the last ~45 kyr, ranging from ~250 to 350 km of ice advance beyond its present position during the LGM to ~20–40 km ice retreat behind its present extent during MIS 3 and ~70 km during the mid-Holocene. During the last ~45 kyr, the NEGIS was smaller than present at least half of the time. These observations present two scenarios that can drive NEGIS retreat within its current extent. Over the early Holocene, the instability of the largely marine-based NEGIS margin was triggered by a combination of air and ocean temperatures similar to today or within the projected scenarios (RCP4.5 to RCP8.5) for the end of this century (Fig. 2d). In contrast, the NEGIS retraction during MIS 3 was potentially due to a combination of lower accumulation/ice flow, elevated incident shortwave radiation, and attendant summer air temperature warming. Our results demonstrate that the NEGIS has responded sensitively to past climatic changes and that its current extent is an anomaly rather than the norm for the last ~45 kyr. These new geologic observations suggest that the NEGIS will continue to undergo ice-margin retreat and lose mass given the ongoing Arctic warming^40^ combined with the topographic setting of large deep fjords^41^ that allow subsurface water to reach and destabilize the ice front^4,5,7^.

## Methods

### Study area

The study area comprises the northern part of the East Greenland Caledonides and the bedrock is primarily composed of crystalline basement except for a few places on Lambert Land and north of Nioghalvfjerdsfjorden, where it is overlain by Paleoprotozoic or Proterozoic sediments^42^. Topographic relief ranges between 0 and 500 m but locally mountains are up to ~1000 m high. The landscape is characterized by selective linear erosion; signs of glacial erosion are significant, particularly at lower elevations. During the LGM, the ice sheet advanced on to the continental shelf in Northeast Greenland^43–45^, but it has been contentious whether it reached the shelf edge ~250–350 km from the present ice margin or remained on the inner shelf (Fig. 1a)^8^. High-resolution multibeam swath bathymetry and shallow seismic data from the shelf offshore NEGIS show a number of glacial landforms including mega-scale glacial lineations, suggesting that the ice extended all the way the shelf edge in Northeast Greenland^9–12^. According to the existing ^14^C-based deglaciation chronology, the outer coast was deglaciated ~9.7–9.5 ka^27^. Following the deglaciation, the land was inundated with marine limits of 40–60 m above sea level^46^.

### Cosmogenic exposure dating

In 2015 and 2016 we conducted fieldwork using helicopter and twin otter plane. We selected field sites using aerial and satellite imagery. Most samples were collected from boulders perched on ice scoured bedrock, except for three samples from Kap Amelié collected from boulders on drift and on Lambert Land where we collected two boulder samples on a moraine outside the Little Ice Age moraine (Fig. 1b). We aimed at sampling glacially abraded boulders on bedrock (Supplementary Fig. 1), with boulders >1 m in height and diameter to reduce the influence of snow cover on our resulting ages^47^. We measured shielding, and recorded the latitude and longitude and elevation using a handheld GPS with an uncertainty of <10 m. The boulder samples were collected using a rock saw. All samples were prepared using carrier “PHE1601” and were measured using the beryllium standard 07KNSTD^48^ at Aarhus AMS Centre (AARAMS). We used the CRONUS-Earth online calculator^49^, the Arctic production rate^50^, and time invariant scaling of Lal/Stone^51,52^ to calculate sample ages (Supplementary Table 1, Table 2). In addition, we used a rock density of 2.7 g cm^−3^ and made no correction for potential snow cover, and surface erosion. The study area has undergone glaciostatic uplift since the deglaciation of ~40 m at Blåsø and ~60 m at Hovgaard Ø^46^, and the sample elevation at the time of collection does not reflect its time-averaged sample elevation history. We calculated sample-specific elevation corrections^25^ and found that the uplift corrections are within 1-sigma of the AMS sample uncertainties similar to or lower than what have been calculated for West Greenland where the postglacial uplift was larger^24,25^. As the uplift history in Northeast Greenland is less well constrained compared to West Greenland^46^ and the vertical uncertainty of the GPS measurement is of the same order as the uplift correction, we present ^10^Be ages without correcting for glaciostatic uplift, similar to most other ^10^Be studies from Greenland^19,20,22,23,53,54^. We note that the lack of this correction does not significantly change our ^10^Be ages or our interpretations. Individual ^10^Be ages are presented with their 1-sigma analytical uncertainties, which include the uncertainty in the blank correction. When we compare our ^10^Be ages with other climate records we include the production rate uncertainty using the following formula:

$$\begin{array}{l}{\mathrm{Uncertainty}} = \\ \sqrt {\left( {1\sigma \,{\mathrm{std}}\,{\mathrm{error}}\,{\mathrm{of}}\,{\mathrm{mean}}} \right)^2 + \left({\,}^{10}{\mathrm{Be}}\,{\mathrm{age}} \times {\mathrm{production}}\,{\mathrm{rate}}\,{\mathrm{uncertainty}}\right)^2} \end{array}$$

$$\begin{array}{l}{\mathrm{Uncertainty}}\,{\mathrm{of}}^{10}{\mathrm{Be}}\,{\mathrm{ages}},{\mathrm{outer}}\,{\mathrm{coast}} = \\ \sqrt {(0.4)^2 + \left( {11.7 \ast 0.037} \right)^2} = 0.6\,{\mathrm{kyr}},\end{array}$$

where 0.4 = 1*σ* standard error of the mean (in kyr), 11.7 = mean ^10^Be age (in ka); 0.037 = the uncertainty associated with the Arctic production rate^50^ and “St” scaling^51,52^.

$$\begin{array}{l}{\mathrm{Uncertainty}}\,{\mathrm{of}}\,^{10}{\mathrm{Be}}\,{\mathrm{ages}},{\mathrm{present}}\,{\mathrm{ice}}\,{\mathrm{margin}} = \\ \sqrt {(0.2)^2 + \left( {9.3 \ast 0.037} \right)^2} = 0.4\,{\mathrm{kyr}},\end{array}$$

where 0.2 = 1*σ* standard error of the mean (in kyr); 9.3 = mean ^10^Be age (in ka), 0.037 = the uncertainty associated with the Arctic production rate^50^ and “St” scaling^51,52^.

We excluded two outliers based on the most general knowledge of the regions glacial history that are older than the LGM (GL1519, GL1545) and most likely contain ^10^Be inherited from a previous period of exposure^47^.

### Radiocarbon dating

A number of shell fragments were collected on the surface of a moraine outside the Little Ice Age moraine on Lambert Land. The shell fragments were identified to species level, when possible. Only large pieces from a single specimen were used for dating. In the laboratory, shell fragments were cleaned and leached using HCl removing c. 25% of the outer shell. Around 10 mg of material was used for the ^14^C analysis; all contained enough carbon for AMS radiocarbon measurement. All radiocarbon ages have been calibrated into calendar years using IntCal13^55^ and a reservoir age of 550 years (∆*R* = 150)^56^ in Oxcal 4.3^57^. The ^14^C ages are reported with 2-sigma uncertainty (Supplementary Table 3).

### Temperature reconstruction

Greenland ice cores provide detailed records on the timing and magnitude of past mean-annual temperature change. However, GrIS mass loss occurs during the summer months^58^, and therefore summer (JJA) temperatures are more relevant than mean-annual temperatures when considering past margin positions. For the last 22 kyr Greenland ice core δ^15^N-based temperature reconstructions^59^ were merged with climate model simulations^60–62^ to generate Greenland-wide, seasonally resolving temperature reconstructions^31^. Supplementary Fig. 2a shows the reconstructed mean-annual (ANN), summer (JJA), and winter (DJF) temperatures at our study location (79^o^N, 20^o^W). Coupled ocean atmosphere GCM simulations are not available through MIS 3; therefore the same approach cannot be used to investigate summer temperatures during that time period. Instead, we rely on a multiple regression approach in which it is assumed that three key forcings dominate the Greenland temperature evolution: AMOC strength, greenhouse gas radiative forcing, and local orbital forcing. Summer temperature at the site (*T*_JJA_) is then given by:

1 $$T_{\mathrm{JJA}} = a_1 \times {\mathrm{AMOC}} + a_2 \times 5.35\ln \frac{{p_{{\mathrm{CO}}_2}}}{280} + a_3 \times {\mathrm{\Phi}}_{\mathrm{JJA}}^{79^\circ {\mathrm{N}}},$$

where AMOC denotes the estimated strength of the overturning in Sv, $p_{{\mathrm{CO}}_2}$ is the atmospheric CO_2_ dry mixing ratio in ppm, ${\mathrm{\Phi }}_{{\mathrm{JJA}}}^{79^\circ {\mathrm{N}}}$is the average solar insolation at 79°N north during the months June, July, and August, and *a*_1_ through *a*_3_ are linear scaling coefficients. The CO_2_ mixing ratio is converted to radiative forcing using the approach by ref. ^63^, with a pre-industrial reference concentration of 280 ppm. All forcings are shown in Supplementary Figs. 2B-D. In reconstructing *T*_JJA_, we use a multi-ice core $p_{{\mathrm{CO}}_2}$ compilation^64^, and insolation values calculated following ref. ^65^. The AMOC strength is the most uncertain of the three forcings and is reconstructed as follows. We start from the Greenland Summit^66,67^ δ^18^O record (average of GRIP and GISP2 δ^18^O records) corrected for mean ocean δ^18^O^68^, and convert it to site (mean annual) temperature using an effective isotope sensitivity of *α* = 0.29 ‰ K^−1^^59^. Using the logic underlying Eq. 1, we remove the effect of CO_2_ forcing (*b*_2_ = 3.05 Km^2^ W^−1^) and insolation (*b*_3_ = 0.0481 Km^2^ W^−1^ sensitivity to local summer insolation) from the GISP2 temperatures, where the stated (annual mean) sensitivities were obtained from the single-forcing deglacial GCM simulations of ref. ^61^, in which greenhouse gas and orbital forcings were applied separately. It is then assumed that remaining temperature variability is due solely to AMOC variability, which we estimate using a sensitivity (mean annual) of *b*_1_ = 1.07 ± 0.25 K Sv^−1^, which optimizes the correlation to the reconstruction by ref. ^31^, as shown in Supplementary Fig. 2B. The NEGIS MIS 3 summer temperature anomaly is then estimated with Eq. 1 using coefficients *a*_1_ = 0.238 ± 0.1 K Sv^−1^, *a*_2_ = 3.76 ± 1.5 Km^2^ W^−1^, and *a*_3_ = 0.137 ± 0.03 Km^2^ W^−1^; these coefficients are found using multiple regression analysis on the 0-22ka NEGIS JJA reconstruction; the coefficients are in good agreement with those found from the single-forcing coupled GCM experiments of ref. ^61^. The MIS 3 JJA reconstruction is shown in Supplementary Fig. 2e, together with an uncertainty envelope that was found by adding all stated uncertainties in quadrature. We want to emphasize the large uncertainty inherent in trying to reconstruct both the AMOC and NEGIS summer temperature based on regression techniques—the resulting values should be considered an order-of-magnitude estimate.

### Data availability

The data that support the findings of this study are available in the supplementary information or it can be acquired from the corresponding author on request.

## Electronic supplementary material

Supplementary Information
