# Supplementary material for: Instability of the Northeast Greenland Ice Stream over the last 45,000 years
Source: Nat Commun. 2018 May 14;9:1872. doi: 10.1038/s41467-018-04312-7 (PMC5951810; doi:10.1038/s41467-018-04312-7)
Supplement: Supplementary file 1 — Supplementary Information [file 41467_2018_4312_MOESM1_ESM.pdf]

Supplementary information

Instability of the Northeast Greenland Ice Stream over the last 45.000 years

Nicolaj K. Larsen[1,2*], Laura B. Levy[3], Anders E. Carlson[4], Christo Buizert[4], Jesper Olsen[5], Astrid Strunk[1], Anders A. Bjørk[2,6], and Daniel S. Skov[1]

[1]Department of Geoscience, Aarhus University, Aarhus, Denmark.

[2]Centre for GeoGenetics, Natural History Museum, University of Copenhagen, Copenhagen, Denmark.

[3]Department of Geology, Humboldt State University, Arcata, California, USA

[4]College of Earth, Ocean, and Atmospheric Sciences, Oregon State University, Corvallis, Oregon, USA

[5]Department of Physics and Astronomy, Aarhus University, Aarhus, Denmark

[6]Department of Earth System Science, University of California, Irvine, California, USA

*Correspondence to: nkl@geo.au.dk

Supplementary figure 1. Pictures of selected sample sites in Northeast Greenland.

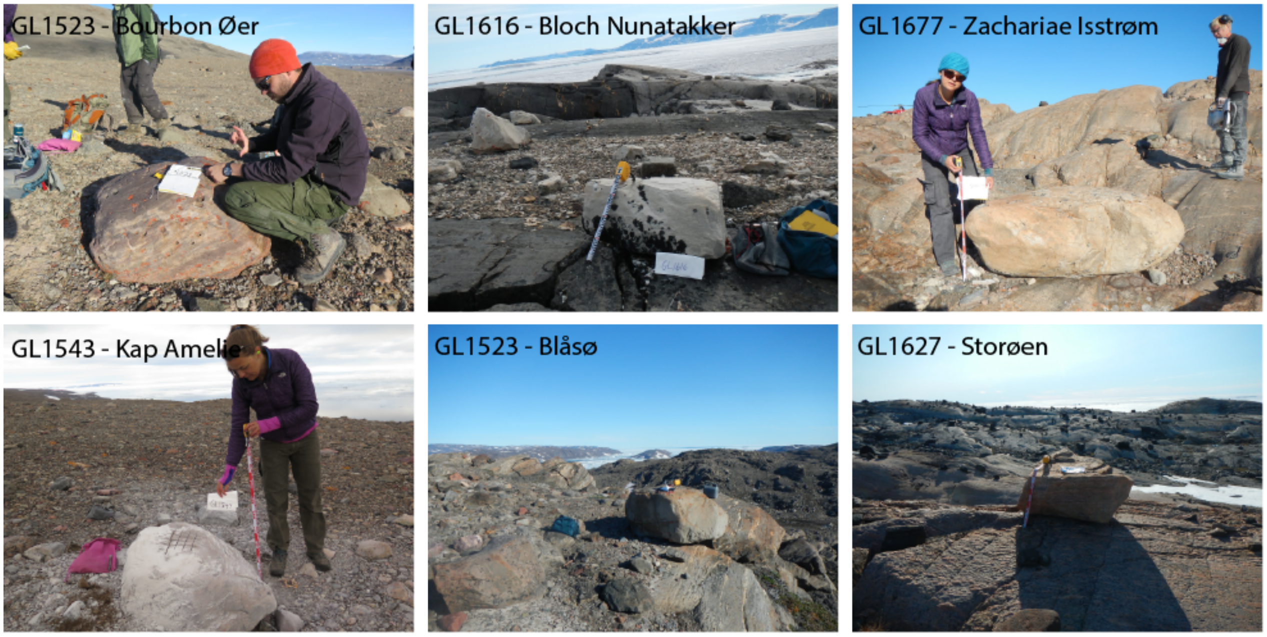

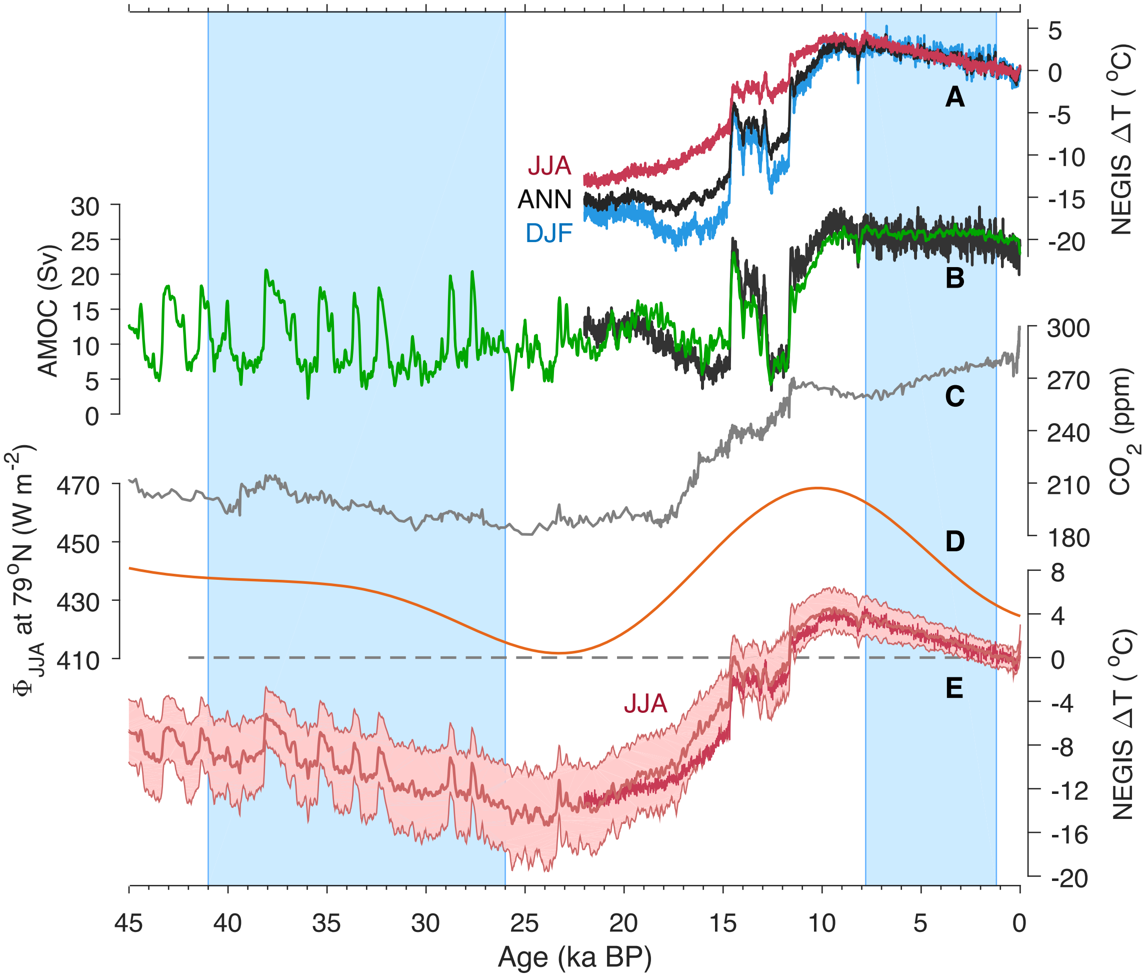

Supplementary figure 2. Reconstructing MIS 3 JJA temperatures using multi-regression analysis. A) NEGIS temperature during summer (JJA), winter (DJF) and mean annual (ANN) from Ref[1]; values given as anomalies relative to present. B) AMOC strength suggested by Ref[1] in black, with AMOC variations used here in green. C) CO_2 concentrations from the compilation by Ref[2]. D) Summer (JJA) insolation at 79°N. E, NEGIS summer temperature from panel (A) in red, with multi-regression results in coral pink with uncertainty envelope.

Supplementary table 1. ^{10}Be ages and associated metadata. We calculated all ^{10}Be ages using the CRONUS-Earth online calculator[6] with the Arctic production rate[3], time invariant scaling ("St") of Lal/Stone[4,5], erosion rate of 0 cm yr^{-1} and a rock density of 2.7 g cm^{-3}. We prepared samples using carrier "PHE1601", which has a measured concentration of 320.6 ppm. Outliers are shown in italics.

Sample ID	Latitude (°N)	Longitude (°W)	Elevation (masl)	Shielding Correction	Thickness (cm)	Quartz (g)	10Be carrier added (g)	10Be Conc. (atoms/g)	Uncertainty (atoms/g)	Age ± Uncertainty (ka)
**Outer coast sites**										
Bourbon Øer: boulders on bedrock										
GL1623	78,620	-18,402	160	1	5,3	40,1634	0,78938	5,12E+04	1,86E+03	10.79 ± 0.39
GL1625	78,620	-18,403	153	1	4,6	40,0475	0,79569	5,42E+04	1,64E+03	11.44 ± 0.35
GL1626	78,621	-18,401	168	0,996	3,5	40,0391	0,79675	5,34E+04	1,79E+03	11.03 ± 0.37
Storøen: boulders on bedrock										
GL1627	78,066	-19,091	263	1	5,8	38,2054	0,80514	5,95E+04	1,74E+03	11.30 ± 0.33
GL1628	78,066	-19,091	264	1	3,9	40,0078	0,80212	6,21E+04	4,55E+03	11.60 ± 0.85
GL1629	78,065	-19,092	263	0,999	5,7	40,019	0,76185	5,93E+04	2,20E+03	11.26 ± 0.42
Kap Amelie: boulders on drift										
GL1543	77,544	-19,131	103	0,999	5,3	40,082	0,79365	5,76E+04	3,24E+03	12.97 ± 0.73
GL1544	77,544	-19,131	104	0,999	4,5	40,2641	0,81004	4,63E+04	4,66E+03	10.34 ± 1.04
GL1545	*77,544*	*-19,131*	*102*	*0,999*	*4,9*	*40,0309*	*0,79894*	*8,75E+04*	*5,93E+03*	*19.70 ± 1.34*
GL1546	77,544	-19,133	99	1	3,9	40,2852	0,8107	6,35E+04	2,07E+03	14.20 ± 0.46
									**Mean**	**11.7 ± 0.4**
**Present-day ice margin sites**										
Blåsø: boulders on bedrock										
GL1522	79,636	-23,093	170	0,993	6,3	40,1929	0,80366	4,78E+04	1,94E+03	10.22 ± 0.42
GL1523	79,637	-23,104	190	0,999	5,7	40,3037	0,79522	3,80E+04	1,30E+04	7.86 ± 2.69
GL1524	79,637	-23,102	108	0,999	5,9	40,042	0,79894	5,33E+04	6,47E+03	12.11 ± 1.47
Lambert Land: boulders on bedrock										
GL1518	79,142	-21,381	236	0,996	5,5	40,0195	0,80417	4,72E+04	2,31E+03	9.27 ± 0.46
GL1519	*79,143*	*-21,406*	*192*	*0,999*	*5,9*	*40,0585*	*0,79443*	*1,94E+05*	*6,66E+03*	*40.28 ± 1.40*
GL1520	79,144	-21,412	215	1	5,4	40,1992	0,80855	4,54E+04	2,78E+03	9.08 ± 0.56
GL1521	79,144	-21,410	210	1	6,1	40,0215	0,80259	4,43E+04	3,22E+03	8.96 ± 0.65
Lambert Land: boulder on moraine										
GL1620	79,101	-20,900	92	0,999	2,8	40,0934	0,80151	4,01E+04	2,17E+03	8.99 ± 0.49
GL1622	79,100	-20,889	103	0,999	4	40,1423	0,79462	3,87E+04	2,42E+03	8.65 ± 0.54
Zachariae Isstrøm: boulders on bedrock										
GL1675	78,782	-20,776	86	1	4,2	35,1096	0,79811	3,87E+04	2,17E+03	8.83 ± 0.50
GL1676	78,781	-20,778	86	0,998	3,3	40,055	0,80178	3,77E+04	1,82E+03	8.55 ± 0.41
GL1677	78,782	-20,779	80	0,999	4,1	40,0178	0,79936	4,29E+04	2,08E+03	9.86 ± 0.48
Søndre Mellemland: boulders on bedrock										
GL1539	78,067	-21,505	90	0,99	5,4	40,2053	0,77266	4,66E+04	2,86E+03	10.82 ± 0.67
GL1540	78,067	-21,505	92	0,999	5,2	40,0691	0,82423	3,94E+04	1,97E+03	9.03 ± 0.45
GL1541	78,067	-21,504	88	0,999	5,4	40,1451	0,80707	4,06E+04	2,18E+03	9.36 ± 0.50
Bloch Nunatakker: boulders on bedrock										
GL1614	79,600	-19,523	72	1	4,4	40,1618	0,80377	3,92E+04	1,52E+03	9.07 ± 0.35
GL1616	79,601	-19,524	75	1	2,3	40,0898	0,79741	3,79E+04	1,42E+03	8.59 ± 0.32
GL1617	79,600	-19,525	76	1	1,9	40,146	0,77685	4,08E+04	2,72E+03	9.21 ± 0.62
									**Mean**	**9.3 ± 0.2**

Supplementary table 2. ^{10}Be ages (in years) with external uncertainties, calculated with various scaling schemes[4,5,7-10]. All ^{10}Be ages are calculated using the Arctic production rate[1], erosion rate of 0 cm yr^{-1} and a rock density of 2.7 g cm^{-3}. Sample outliers are italicized.

Scaling scheme	Desilets and others (2003, 2006)		Dunai (2001)		Lifton and others (2005)		Time-dependent Lal (1991)/Stone (2000)		Time-independent Lal (1991)/Stone (2000)	
Sample name	Exposure age (yr)	External uncertainty (yr)	Exposure age (yr)	External uncertainty (yr)	Exposure age (yr)	External uncertainty (yr)	Exposure age (yr)	External uncertainty (yr)	Exposure age (yr)	External uncertainty (yr)
GL1518	9260	600	9240	610	9280	610	9270	580	9270	580
GL1519	*40090*	*2180*	*40000*	*2270*	*39800*	*2230*	*40280*	*2090*	*40280*	*2090*
GL1520	9050	670	9040	690	9070	680	9080	660	9080	660
GL1521	8930	750	8910	760	8940	760	8960	740	8960	740
GL1522	10150	590	10130	610	10190	610	10220	570	10220	570
GL1523	7820	2700	7800	2700	7820	2700	7860	2710	7860	2710
GL1524	11930	1540	11910	1540	12000	1550	12110	1550	12110	1550
GL1539	10640	790	10630	810	10710	810	10820	780	10820	780
GL1540	8890	580	8870	590	8910	590	9030	570	9030	570
GL1541	9210	630	9200	640	9240	640	9360	620	9360	620
GL1543	12790	900	12780	920	12850	920	12970	890	12970	890
GL1544	10200	1110	10190	1120	10260	1130	10340	1120	10340	1120
GL1545	19430	1550	19390	1580	19410	1570	19700	1540	19700	1540
GL1546	13990	740	13970	770	14040	770	14200	720	14200	720
GL1614	8900	510	8890	530	8930	520	9070	490	9070	490
GL1616	8440	470	8420	490	8450	490	8590	460	8590	460
GL1617	9040	710	9030	730	9070	730	9210	710	9210	710
GL1620	8850	610	8840	620	8870	620	8990	600	8990	600
GL1622	8530	640	8520	650	8540	650	8650	640	8650	640
GL1623	10710	590	10690	610	10760	610	10790	570	10790	570
GL1625	11350	590	11330	610	11410	610	11440	560	11440	560
GL1626	10960	590	10940	610	11020	610	11030	560	11030	560
GL1627	11310	580	11290	600	11350	600	11300	540	11300	540
GL1628	11610	980	11590	1000	11660	1000	11600	960	11600	960
GL1629	11270	630	11250	650	11320	650	11260	600	11260	600
GL1675	8680	610	8670	620	8700	620	8830	600	8830	600
GL1676	8410	540	8400	550	8430	550	8550	530	8550	530
GL1677	9680	620	9670	640	9730	640	9860	610	9860	610

Supplementary table 3. Compilation of radiocarbon dates from Northeast Greenland: Lambert Land (this study), Blåsø, Midgaardsormen and Søndre Mellemland[11], and Storstrømmen[12].

Location	Latitude	Longitude	Altitude (m a.s.l.)	Material	Lab No.	Age (14C BP)	Max calibrated age (cal. yr BP)	Min calibrated age (cal. yr BP)	Reference
Lambert Land	79° 6.0'	20° 53.4'	77	Hiatella arctica	AAR-26187	37079±582	42105	40011	This study
Lambert Land	79° 6.0'	20° 53.4'	77	Shell fragments (snail)	AAR-26189	35353±195	39833	38793	This study
Lambert Land	79° 6.0'	20° 53.4'	77	Shell fragments (suspension feeder)	AAR-26183	35240±464	40328	38347	This study
Lambert Land	79° 6.0'	20° 53.4'	77	Shell fragments (snail)	AAR-26188	31318±199	35088	34260	This study
Lambert Land	79° 6.0'	20° 53.4'	77	Shell fragments (suspension feeder)	AAR-26182	31216±421	35480	33915	This study
Lambert Land	79° 6.0'	20° 53.4'	77	Mya truncata	AAR-26185	30788±297	34780	33793	This study
Lambert Land	79° 6.0'	20° 53.4'	77	Hiatella arctica	AAR-26186	24405±215	28419	27589	This study
Lambert Land	79° 6.0'	20° 53.4'	77	Astarte borealis	AAR-26184	22602±139	26620	25959	This study
Blåsø	79°34.6'	22°37.3'	11	?Pusa hispida	AAR-4693	6940±75	7441	7163	11
Blåsø	79°34.9'	22°18.2'	7	?Pusa hispida	AAR-3838	6755±55	7252	6980	11
Blåsø	79°37.6'	21°55.5'	27	Larix sp.	AAR-4121	6080±55	7156	6795	11
Blåsø	79°36.8'	22°30.9'	0,5	Larix sp.	AAR-4128	6040±55	7151	6739	11
Blåsø	79°37.6'	22°23.0'	24	?Larix sp.	AAR-3835	6035±55	7150	6736	11
Blåsø	79°36.8'	22°30.9'	1	Larix sp.	AAR-3836	5995±55	6970	6679	11
Blåsø	79°37.2'	22°35.0'	1	Larix sp.	AAR-3837	5955±55	6930	6666	11
Blåsø	79°40'	22°30'	1	?Larix sp.	AAR-4129	5440±55	6393	6020	11
Blåsø	79°37.6'	22°23.0'	5	Larix sp.	AAR-3834	5080±55	5928	5664	11
Blåsø	79°34.6'	22°37.3'	11	?Pusa hispida	AAR-4215	5585±65	5941	5643	11
Blåsø	79°34.3'	22°23.3'	19-20	?B. mysticetus	K-6891	5190±70	5566	5262	11
Blåsø	79°32.9'	22°37.4'	1	?Pusa hispida	AAR-4216	4645±55	4820	4525	11
Blåsø	79°36.8'	22°30.9'	9	?Pusa hispida	AAR-3833	4590±45	4790	4480	11
Midgaardsormen	79°35.2’	21°40.9’	20	Hiatella arctica	Ua-10557	7480±170	8149	7494	11
Midgaardsormen	79°39.7’	21°05.2’	12	Astarte borealis	Ua-11480	7045±75	7546	7266	11
Midgaardsormen	79°32.3'	22°26.6'	33	Mya truncata	AAR-3839	6805±65	7320	7001	11
Midgaardsormen	79°39.7’	21°05.2’		Populus	Ua-11481	6160±75	7254	6860	11
Midgaardsormen	79°39.8’	21°02.6’	30	Mya truncata	Ua-11479	6665±70	7200	6845	11
Midgaardsormen	79°39.7’	21°02.8’	20	Mya truncata	Ua-11478	6535±80	7085	6660	11
Midgaardsormen	79°38.9’	21°09.0’	11	Mya truncata	Ua-11484	6245±80	6712	6336	11
Midgaardsormen	79°38.9’	21°09.2’	11	Hiatella arctica	Ua-11482	6310±75	6589	6256	11
Midgaardsormen	79°38.9’	21°09.9’	11	Hiatella arctica	Ua-11485	6125±80	6596	6243	11
Midgaardsormen	79°38.9’	21°09.0’	17?	Mya truncata	Ua-11483	6115±75	6565	6232	11
Midgaardsormen	79°38.9’	21°09.0’	17?	Hiatella arctica	Ua-11486	6065±80	6513	6173	11
Midgaardsormen	79°36.8'	22°30.9'	32	Mya truncata	K-6894	6050±105	6580	6090	11
Midgaardsormen	79°39.5’	21°08.8’	10	Mya truncata	Ua-10553	5985±65	6397	6106	11
Midgaardsormen	79°39.5’	21°01.8’	10	Mya truncata	Ua-10552	5945±70	6363	6018	11
Midgaardsormen	79°34.4'	22°23.3'	7	Serripes groenlandicus	AAR-4699	5830±50	6210	5940	11
Midgaardsormen	79°36.6'	22°18.3	12	Serripes groenlandicus	AAR-4120	5775±60	6175	5896	11
Midgaardsormen	79°39.5’	21°01.8’	10	Mya truncata	Ua-10554	5695±80	6153	5739	11
Midgaardsormen	79°35.2’	21°40.9’	20	Mya truncata	Ua-10556	5660±90	6129	5670	11
Midgaardsormen	79°39.5’	21°01.8’	10	Hiatella arctica	Ua-10555	5405±85	5843	5446	11
Midgaardsormen	79°39.0'	21°07.2'	10	Picea sp.	K-6893	4730±95	5657	5085	11
Midgaardsormen	79°39.6’	21°03.0’	12	Hiatella arctica	Ua-11477	5135±70	5535	5117	11
Midgaardsormen	79°38.9'	21°08.1'	15	Serripes groenlandicus	AAR-4127	4965±55	5278	4928	11
Søndre Mellemland (Sanddalen)	78°4.6'	21°38'	90	Astarte borealis	AAR-5053	5875±60	6270	5982	11
Søndre Mellemland (Sanddalen)	78°4.6'	21°42'	80	Astarte borealis	AAR-5055	5750±60	6165	5880	11
Søndre Mellemland (Sanddalen)	78°4.6'	21°38'	90	Astarte borealis	AAR-5054	5630±60	5992	5698	11
Søndre Mellemland (Sanddalen)	78°4.6'	21°58'	200	Hiatella arctica	AAR-5056	5550±55	5895	5640	11
Søndre Mellemland (Sanddalen)	78°4.6'	21°58'	200	Hiatella arctica	AAR-5057	5500±55	5859	5594	11
Søndre Mellemland (Sanddalen)	78°04.6'	21°42'	80	Hiatella arctica	AAR-4701	5350±55	5682	5442	11
Storstrømmen	c77°10’	21°58’		Hiatella arctica	Ua-3348	1815±55	1327	1091	12
Storstrømmen	77°5.1’	21°55.2’	13	Hiatella arctica/Mya truncata	K-6097	3230±85	3090	2702	12
Storstrømmen	77°9.8’	21°58.7’	135-150	Balaenoptera	K-6096	3630±90	3585	3130	12
Storstrømmen	c77°10’	21°58’		Hiatella arctica	Ua-3349	3725±60	3620	3334	12
Storstrømmen	c77°10’	21°55’		Hiatella arctica	Ua-3350	4180±60	4231	3872	12
Storstrømmen	77°11’	21°57’	150	Mya truncata	K-5493	4840±90	5244	4714	12
Storstrømmen	77°11’	21°57’	150	Mya truncata	K-5494	4910±85	5264	4830	12
Storstrømmen	c77°10’	22°0’		Hiatella arctica	Ua-3347	5030±75	5410	4959	12
Storstrømmen	77°9.9’	21°58.7’	140	Hiatella arctica/Mya truncata	K-6098	5180±95	5583	5135	12
Storstrømmen	c77°10’	c22°		Astarte borealis	Ua-275	24930±275	28955	27834	12
Storstrømmen	c77°10’	c22°		Mya truncata	Ua-4570	26665±300	30931	29618	12
Storstrømmen	c77°10’	c22°		Hiatella arctica	Ua-4569	27905±370	32142	30769	12
Storstrømmen	c77°10’	c22°		Hiatella arctica	Ua-3352	33250±815	38899	35064	12

References

1 Buizert, C. *et al.* Greenland-wide seasonal temperature reconstructions for the last deglaciation. *Physical Review Letters* 45, 1905–1914, doi:10.1002/2017GL075601 (2018)

2 Bereiter, B. *et al.* Revision of the EPICA Dome C CO2 record from 800 to 600 kyr before present. *Geophys. Res. Lett.* **42**, 2014GL061957, doi:10.1002/2014gl061957 (2015).

3 Young, N. E., Schaefer, J. M., Briner, J. P. & Goehring, B. M. A Be-10 production-rate calibration for the Arctic. *Journal of Quaternary Science* **28**, 515-526, doi:10.1002/Jqs.2642 (2013).

4 Lal, D. Cosmic-ray labelling of erosion surfaces: in situ nuclide production rates and erosion models. *Earth and Planetary Science Letters* **104**, 424-439 (1991).

5 Stone, J. O. Air pressure and cosmogenic isotope production. *Journal of Geophysical Research* **105 (B10)**, 23753-23759 (2000).

6 Balco, G., Stone, J. O., Lifton, N. A. & Dunai, T. J. A complete and easily accessible means of calculating surface exposure ages or erosion rates from Be-10 and Al-26 measurements. *Quat Geochronol* **3**, 174-195, doi:10.1016/j.quageo.2007.12.001 (2008).

7 Desilets, D. & Zreda, M. Spatial and temporal distribution of secondary cosmic-ray nucleon intensities and applications to in situ cosmogenic dating. *Earth and Planetary Science Letters* **206**, 21-42, doi:Pii S0012-821x(02)01088-9. Doi 10.1016/S0012-821x(02)01088-9 (2003).

8 Desilets, D., Zreda, M. & Prabu, T. Extended scaling factors for in situ cosmogenic nuclides: New measurements at low latitude. *Earth and Planetary Science Letters* **246**, 265-276, doi:10.1016/j.epsl.2006.03.051 (2006).

9 Dunai, T. J. Influence of secular variation of the geomagnetic field on production rates of in situ produced cosmogenic nuclides. *Earth and Planetary Science Letters* **193**, 197-212, doi:Doi 10.1016/S0012-821x(01)00503-9 (2001).

10 Lifton, N. A. *et al.* Addressing solar modulation and long-term uncertainties in scaling secondary cosmic rays for in situ cosmogenic nuclide applications. *Earth and Planetary Science Letters* **239**, 140-161, doi:10.1016/j.epsl.2005.07.001 (2005).

11 Bennike, O. & Weidick, A. Late Quaternary history around Nioghalvfjerdsfjorden and Jokelbugten, North-East Greenland. *Boreas* **30**, 205-227 (2001).

12 Weidick, A., Andreasen, C., Oerter, H. & Reeh, N. Neoglacial glacier changes around Storstrømmen, North-East Greenland. *Polarforschung* **64**, 95-108 (1996).
